# Fundamental solution of the time-space bi-fractional diffusion equation with a kinetic source term for anomalous transport

**DOI:** 10.1038/s41598-024-63579-7

**Published:** 2024-06-05

**Authors:** Anis Allagui, Georgii Paradezhenko, Anastasiia Pervishko, Dmitry Yudin, Hachemi Benaoum

**Affiliations:** 1 Department of Sustainable and Renewable Energy Engineering, University of Sharjah, P.O. Box 27272 Sharjah, United Arab Emirates; 2 Department of Mechanical and Materials Engineering, Florida International University, Miami, FL 33174 USA; 3 Skolkovo Institute of Science and Technology, Moscow, 121205 Russia; 4 Department of Applied Physics and Astronomy, University of Sharjah, PO Box 27272 Sharjah, United Arab Emirates

**Keywords:** Applied mathematics, Chemical physics

## Abstract

The purpose of this paper is to study the fundamental solution of the time-space bi-fractional diffusion equation incorporating an additional kinetic source term in semi-infinite space. The equation is a generalization of the integer-order model ${\partial _{{t}} {\rho (x,t)}} = {\partial ^2_{{x}} {\rho (x,t)}} - { \rho (x,t)}$ (also known as the Debye–Falkenhagen equation) by replacing the first-order time derivative with the Caputo fractional derivative of order $0<\alpha < 1$, and the second-order space derivative with the Riesz-Feller fractional derivative of order $0< \beta <2$. Using the Laplace-Fourier transforms method, it is shown that the parametric solutions are expressed in terms of the Fox’s *H*-function that we evaluate for different values of $\alpha$ and $\beta$.

## Introduction

The general reaction-diffusion equation in a normalized form can be written as^1^:

1 $$\begin{aligned} {\partial _{{t}} {\rho }} = {\partial ^2_{{x}} {\rho }} +f(\rho ) \end{aligned}$$

where $\rho = \rho (x,t)$ is the field variable that depends on position *x* and time *t*, and the functional $f(\rho )$ is usually a nonlinear term pertinent to the process under consideration (e.g. $f(\rho )=\rho (1-\rho )$ for Kolmogorov, Petrovsky, and Piskunov (KPP) nonlinearity, $f(\rho )=\rho ^m (1-\rho )$ for the $m^{\text {th}}$-order Fisher nonlinearity, etc.). For the particular case of $f(\rho )=-\rho$ we end up with the Debye–Falkenhagen equation:

2 $$\begin{aligned} {\partial _{{t}} {\rho }} = {\partial ^2_{{x}} {\rho }} -\rho \end{aligned}$$

which is used to describe a classical mean-field problem of electrodiffusive transport in electrochemistry (see Janssen^2^, Janssen and Bier^3^, Bazant et al.^4^, Singh and Kant^5–7^, and many others^8^). This model is best used for describing dynamics at planar electrodes. The partial differential equation given in (2) may be looked upon as a analog of the standard diffusion equation with a kinetic source term^5^. However, in practice, electrochemical devices and systems unavoidably exhibit in a way or another anomalies in their electrical response and frequency dispersion of their properties due to their structural disorder, spatial heterogeneity, and wide spectrum of relaxation times.

Specifically, Eq. (2) considers changes in the reduced density of charge (the field variable $\rho (x,t)$) through a control volume to be linear and memoryless, due to the fact that we only use a first-order Taylor series approximation in space and time^9^. Differential equations with integer-order differential operator are actually defined in an infinitesimally small neighborhood of the point under consideration, and therefore are a tool for describing only local media^10^. For the case of non-local media, the size of the control volume must be large enough compared to the scale(s) of the heterogeneity in the medium, which makes integer-order derivatives inadequate for describing media with heterogeneity. Furthermore, spatial heterogeneities are not necessarily static in the course of operation of the device or system, and therefore memory effects shall be taken into consideration^11^. This renders the problem of studying the complex behavior of electrochemical systems restricted when using traditional integer-order models^12–16^.

For a proper theoretical modeling of anomalous transport, one can adopt fractional calculus to include fractional time and/or spacial derivatives^17^. This is mainly attributed to the fact that the dynamics of transport processes substantially differs from the picture of classical transport owing to memory effects or spatial nonlocality of purely non-Markovian nature. Fractional calculus permits to deal with such situations via integrals and derivatives of any arbitrary real or complex order, and therefore permits to unify and extend integer-order integrals and derivatives used in classical models^11,18,19^. Saichev and Zaslavsky^20^, Mainardi et al.^11^, Gorenflo et al.^21^, and Bologna^22,23^ studied the generalization of the standard diffusion equation with fractional derivatives with respect to time and space (i.e. $\partial _t\rho = \partial _x^2 \rho$), in which the first-order time derivative of the propagating quantity was replaced with a Caputo derivative and the second-order space derivative was replaced with a Riesz-Feller derivative (i.e. $^\text{c}D_t^\alpha \rho =D_{x}^{\beta ,\theta }\rho$, see definitions below). Kosztołowicz and Metzler^24^ described the transport of an antibiotic in a biofilm using a time-fractional subdiffusion-absorption equation based on the Riemann- Liouville time-fractional derivative. Saxena, Mathai and Haubold studied extensively in a series of papers^25–29^ unified forms of fractional kinetic equations and fractional reaction-diffusion equations in which the time derivative is replaced by either the Caputo, Riemann-Liouville or a generalized fractional derivative as defined by Hilfer^30^, and the space derivative is replaced by the Riesz-Feller derivative. Additional nonlinear terms pertinent to reaction processes are also considered. Fractional reaction-diffusion equations are of specific interest in a large class of science and engineering problems for describing non-Gaussian, non-Markovian, and non-Fickian phenomena.

The goal of this work is to study the bi-fractional (time and space) generalization of the (dimensionless) diffusion equation with a kinetic source term of the Debye and Falkenhagen type (see Section “Model”, Eq. (3) below), and understand how do the fractional orders of differentiation affect the dynamics of the propagating quantity. In Section “Analytical solutions” we provide the analytical solution to this equation in terms of Fox’s *H*-function using integral transform methods (Laplace–Fourier), followed by numerical simulations in Section “Numerical results” for different sets of values for the fractional parameters.

## Model

We consider the bi-fractional diffusion equation with an additional source term given in one dimension by:

3 $$\begin{aligned} {}^\text{c}D_t^\alpha \rho =D_{x}^{\beta ,\theta }\rho -\rho \end{aligned}$$

subjected to the boundary and initial conditions

4 $$\begin{aligned} \rho (x=\pm \infty ,t) = 0, \qquad \rho (x,t=0) = \delta (x). \end{aligned}$$

This model is a generalization of Eq. (2) (i.e. by setting $\alpha =1$, $\beta =2$, $\theta =0$ in Eq. (3)), and can describe for example the situation of anomalous ion transport through electrified membranes, porous electrodes, or other complex systems. In Eq. (3), the operator $^\text{c}D^{\alpha }_t$ is the Caputo time fractional derivative of order $\alpha$ ($0< \alpha < 1$) replacing the first order time derivative in Eq. (2), and $D^{\beta ,\theta }_x$ is the Riesz-Feller space fractional derivative of order $\beta$ ($0< \beta < 2$) replacing the second order space derivative^11^. The Caputo time-fractional derivative of order $\alpha$ ($m-1<\alpha <m, m\in \mathbb {N}$) of *f*(*t*) is defined through the Laplace transform ($\tilde{f}(s) = \mathcal {L}[f(t);s] = \int _0^{\infty } e^{-st} f(t) dt, s\in \mathbb {C}$) by:

5 $$\begin{aligned} \mathcal {L}\left\{ ^\text{c}D^{\alpha }_t f(t); s \right\} = s^{\alpha } \tilde{f}(s) - \sum _{r=0}^{m-1} s^{\alpha - r - 1} f^{(r)}(0), \end{aligned}$$

This lead to the integro-differential definition:

6 $$\begin{aligned} {}^\text{c}D^{\alpha }_t f(t) \equiv \frac{1}{\Gamma (m-\alpha )} \int \limits _0^t \frac{f^{(m)}(\tau ) d\tau }{(t-\tau )^{\alpha +1-m}}, \end{aligned}$$

that takes into account all past activities of the function up to the current time. For the case of $\alpha =m$, we have the traditional, memoryless integer-order derivative:

7 $$\begin{aligned} {}^\text{c}D^{\alpha }_t f(t) = \frac{d^m f(t)}{dt^m} \end{aligned}$$

Whereas for a sufficiently well-behaved function *f*(*x*), the Riesz-Feller space-fractional derivative of order $\beta$ ($0<\beta \leqslant 2$) and skewness $\theta$ ($|\theta | < \min \left\{ \beta , 2-\beta \right\}$) is defined in terms of its Fourier transform ($\hat{f}(k) = \mathcal {F}\{ f(x);k\} = \int _{-\infty }^{\infty } e^{ikx} f(x) dx, k\in \mathbb {R}$) as^11^:

8 $$\begin{aligned} \mathcal {F}\left\{ D^{\beta ,\theta }_x f(x); k \right\} = -|k|^\beta e^{i (\text{sgn} k)\theta \pi /2} \hat{f}(k) \end{aligned}$$

In terms of integral representation, the Riesz-Feller derivative can be represented by:^28^:

9 $$\begin{aligned} D^{\beta ,\theta }_x f(x) = \frac{\Gamma (1+\beta )}{\pi } \times \left\{ \sin \left[ (\beta +\theta )\pi /2 \right] \int \limits _0^{\infty } \frac{f(x+\xi ) - f(x)}{ \xi ^{1+\beta }} d\xi + \sin \left[ (\beta -\theta )\pi /2 \right] \int \limits _0^{\infty } \frac{f(x -\xi ) - f(x)}{ \xi ^{1+\beta }} d\xi \right\} \end{aligned}$$

For the specific case of $\theta =0$, we have the symmetric operator with respect to *x* that can be interpreted as:

10 $$\begin{aligned} D^{\beta ,0}_x f(x) = -\left[ -\frac{d^2}{dx^2}\right] ^{\beta /2} \end{aligned}$$

and Eq. (8) reduces to:

11 $$\begin{aligned} \mathcal {F}\left\{ D^{\beta ,0}_x f(x); k \right\} = -|k|^\beta \hat{f}(k) \end{aligned}$$

We note that there are many other ways to generalize the integer-order time and space derivatives to fractional orders, using for instance the Hilfer/Hilfer-Katugampola and Weyl fractional operators as shown by Costa et al.^31,32^. However, for the boundary and initial conditions given in (4), the classical definitions of Caputo and Riesz-Feller are well suited for this study.

## Analytical solutions

### Case with $0<\alpha < 1$, $\beta =2$

We start with the simple case of $\beta = 2$ and skewness $\theta =0$, which makes Eq. (3) to reduce to the time fractional equation of the form

12 $$\begin{aligned} ^\text{c}D^{\alpha }_t \rho = \partial ^2_{x} \rho - \rho . \end{aligned}$$

Taking into account the Laplace transform of the Caputo fractional time derivative, Eq. (12) in the Laplace space takes the form:

13 $$\begin{aligned} s^{\alpha }\tilde{\rho }(x,s) - s^{\alpha -1} \rho (x,0) = \partial ^2_{x} \tilde{\rho }(x,s) - \tilde{\rho }(x,s). \end{aligned}$$

Using (4) and making the Fourier transform for both sides of Eq. (13), we come to

14 $$\begin{aligned} s^{\alpha }\hat{\tilde{\rho }}(k,s) - s^{\alpha -1} = -k^2 \hat{\tilde{\rho }}(k,s) - \hat{\tilde{\rho }}(k,s). \end{aligned}$$

Thus, the solution of Eq. (12) in the Laplace-Fourier space reads,

15 $$\begin{aligned} \hat{\tilde{\rho }}(k,s) = \frac{s^{\alpha -1}}{s^\alpha + 1 + k^2}. \end{aligned}$$

In general, integral transform methods are commonly used for studying fractional linear diffusion equations, but when nonlinear terms are present other analytical techniques or numerical methods can be applied^33–37^.

#### Solution in the real-Laplace space

To get the solution in the real space, it is convenient to make the inverse Laplace and Fourier transforms with respect to *s* and *k*, sequentially^11^. However, we might be interested in the solution obtained by the inverse Fourier transform with respect to *k* and remained in the Laplace space with respect to time *t*. Formally, one can write this solution in the form

16 $$\begin{aligned} \tilde{\rho }(x,s) = \frac{1}{2\pi } \int \limits _{-\infty }^{\infty } \frac{s^{\alpha -1}}{s^{\alpha } + 1 + k^2} e^{-ixk}\,dk. \end{aligned}$$

Introducing the notation $s^\alpha +1 = a$ ($\text{Re}(s)>0$ and $\text{Re}(a)>0$), we have

17 $$\begin{aligned} \tilde{\rho }(x,s)= & {} \frac{s^{\alpha -1}}{2\pi } \int \limits _{-\infty }^{\infty } \frac{e^{-ixk}\,dk}{a + k^2}\nonumber \\= & {} \frac{s^{\alpha -1}}{2\pi } \int \limits _{-\infty }^{\infty } \frac{e^{-ixk}\,dk}{(k - \sqrt{a}i)(k + \sqrt{a}i)}. \end{aligned}$$

**Figure 1:**
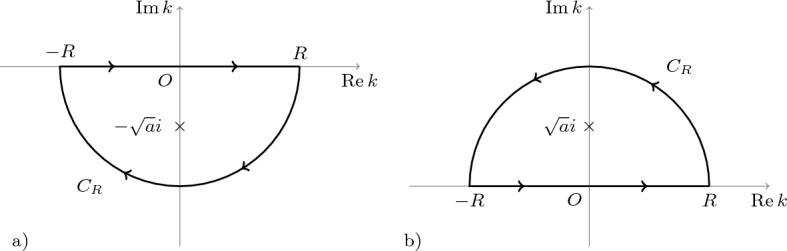
The integration contours for (**a**) $x>0$ and (**b**) $x<0$, and poles of the integrand on the left-hand side for Eq. (17).

The integrand in Eq. (17) is analytic everywhere except for the isolated singularities $k = \pm \sqrt{a} i$, where it has simple poles. For $x>0$, using the residue theorem, we have

18 $$\begin{aligned} \lim _{R \rightarrow \infty } \oint _{C_R} \frac{e^{-i k x} \, dk}{(k - \sqrt{a}i)(k + \sqrt{a}i)} = -2\pi i \, \text{res}_{k = -\sqrt{a}i} \left[ \frac{ e^{-i k x}}{(k - \sqrt{a}i)(k + \sqrt{a}i)}\right] , \end{aligned}$$

where the contour $C_R$ is shown in Fig. 1a.

As $R \rightarrow \infty$, the integral over the arc of the circle tends to zero, because the integrand

$$\begin{aligned} \frac{e^{-i kx}}{(k + \sqrt{a}i)(k - \sqrt{a}i)} = \frac{ e^{-i x\mathop {\text{Re}}k} e^{x\mathop {\text{Im}}k}}{(k + \sqrt{a}i)(k - \sqrt{a}i)}, \quad \mathop {\text{Im}}k < 0 \end{aligned}$$

vanishes exponentially for $x>0$. Therefore,

19 $$\begin{aligned} \lim _{R \rightarrow \infty } \oint _{C_R} \frac{e^{-i k x} \, dk}{(k - \sqrt{a}i)(k + \sqrt{a}i)} = \int \limits _{-\infty }^{\infty } \frac{e^{-i kx} \, dk}{(k + \sqrt{a}i)(k - \sqrt{a}i)}. \end{aligned}$$

Calculating the residue, we obtain

20 $$\begin{aligned} \text{res}_{k = -\sqrt{a}i} \left[ \frac{e^{-i kx}}{(k + \sqrt{a}i)(k - \sqrt{a}i)} \right] = -\frac{e^{-\sqrt{a}x}}{2\sqrt{a}i}. \end{aligned}$$

Substituting the latter and (19) in Eq. (18), we obtain

21 $$\begin{aligned} \int \limits _{-\infty }^{\infty } \frac{e^{-i kx} \, dk}{(k + \sqrt{a}i)(k - \sqrt{a}i)} = \frac{\pi e^{-\sqrt{a}x}}{\sqrt{a}}, \quad x >0. \end{aligned}$$

Thus, Eq. (17) takes the form

22 $$\begin{aligned} \tilde{\rho }(x,s) = \frac{s^{\alpha -1}e^{-\sqrt{a}x}}{2\sqrt{a}}, \quad x > 0. \end{aligned}$$

Similarly, for $x<0$ we consider the contour $C_R$ is shown in Fig. 1b. The result for Eq. (17) in this case reads,

23 $$\begin{aligned} \tilde{\rho }(x,s) = \frac{s^{\alpha -1}e^{\sqrt{a}x}}{2\sqrt{a}}, \quad x < 0. \end{aligned}$$

Then, by combining Eqs. (22) and (23) together, we come to

24 $$\begin{aligned} \tilde{\rho }(x,s) = \frac{s^{\alpha -1}e^{-\sqrt{a}|x|}}{2\sqrt{a}}. \end{aligned}$$

Finally, using $a = s^{\alpha } + 1$, we obtain

25 $$\begin{aligned} \tilde{\rho }(x,s) = \frac{s^{\alpha -1}}{2(s^{\alpha } + 1)^{1/2}} \exp \left[ - |x| (s^{\alpha }+1)^{1/2} \right] . \end{aligned}$$

We should note that for the time-fractional diffusion equation

26 $$\begin{aligned} ^\text{c}D^{\alpha }_t \rho = \partial ^2_{x} \rho , \end{aligned}$$

the solution in the Laplace-Fourier space reads,

27 $$\begin{aligned} \hat{\tilde{\rho }}(k,s) = \frac{s^{\alpha -1}}{s^\alpha + k^2}. \end{aligned}$$

Thus, from Eq. (24) with $a = s^\alpha$, one can get the solution in agreement with^38^,

28 $$\begin{aligned} \tilde{\rho }(x,s) = \frac{1}{2} s^{\alpha /2-1} \exp \left[ -|x| s^{\alpha /2} \right] . \end{aligned}$$

#### Solution in the Fourier-time space

Unfortunately, the inverse Laplace transform of Eq. (25) is problematic. However, we can invert the Laplace transform from Eq. (15) following Langlands^39^. We rewrite Eq. (15) as

29 $$\begin{aligned} \hat{\tilde{\rho }}(k,s) = \frac{s^{\alpha -1}}{s^\alpha + 1 + k^2} = \frac{s^{\alpha -1}}{s^\alpha + k^2} \frac{1}{1 + \frac{1}{s^{\alpha } + k^2}}. \end{aligned}$$

Now by expanding the second fraction we have

30 $$\begin{aligned} \hat{\tilde{\rho }}(k,s) = \frac{s^{\alpha -1}}{s^\alpha + k^2} \sum _{r=0}^\infty \frac{(-1)^r}{(s^\alpha + k^2)^r} = \sum _{r=0}^\infty \frac{(-1)^r s^{\alpha -1} }{(s^\alpha + k^2)^{r+1}}. \end{aligned}$$

From^40^ we have the following Laplace transform

31 $$\begin{aligned} \mathcal {L}\left\{ t^{\alpha r + \beta - 1} E^{(r)}_{\alpha ,\beta }(-at^\alpha ); s \right\} = \frac{r! s^{\alpha -\beta }}{(s^\alpha + a)^{r+1}}, \end{aligned}$$

where

32 $$\begin{aligned} E_{\alpha ,\beta }(z) = \sum _{k=0}^\infty \frac{z^k}{\Gamma (\alpha k + \beta )} \end{aligned}$$

is the Mittag–Leffler function. Thus, using Eq. (31) with $a=k^2$ and $\beta = 1$, we can invert the Laplace transform in (30) to get

33 $$\begin{aligned} \hat{\rho }(k,t) = \sum _{r=0}^\infty \frac{(-1)^r t^{\alpha r} }{r!} E_{\alpha }^{(r)} (-k^2 t^\alpha ). \end{aligned}$$

The derivatives of the Mittag–Leffler function can be expressed in terms of the Fox’s *H*-function, which is defined by means of a Mellin-Barnes type integral in the following manner^39,41–43^:

34 $$\begin{aligned} H^{m,n}_{p,q}(z) = H^{m,n}_{p,q}\left[ z|^{(a_p,A_p)}_{(b_q,B_q)} \right] =H^{m,n}_{p,q}\left[ z|^{(a_1,A_1),\ldots ,(a_p,A_p)}_{(b_1,B_1),\ldots , (b_q,B_q)} \right] =\frac{1}{2\pi i} \int _L h(s) z^{-s} ds \end{aligned}$$

with *h*(*s*) given by the ratio of products of Gamma functions:

35 $$\begin{aligned} h(s) = \frac{ \prod _{j=1}^m \Gamma (b_j + B_j s) \prod _{j=1}^n \Gamma (1-a_j - A_j s) }{ \prod _{j={n+1}}^p \Gamma (a_j + A_j s) \prod _{j={m+1}}^q \Gamma (1-b_j - B_j s) } \end{aligned}$$

*m*, *n*, *p*, *q* are integers satisfying ($0 \leqslant n \leqslant p$, $1 \leqslant m \leqslant q$), $z\ne 0$, and $z^{-s}=\exp \left[ -s (\ln |z|+ i \arg z) \right]$, $A_i, B_j \in \mathbb {R}_+$, $a_i, b_j \in \mathbb {R}$ or $\mathbb {C}$ with $(i=1,2,\ldots ,p)$, $(j=1,2,\ldots ,q)$. The contour of integration *L* is a suitable contour separating the poles $-(b_j+\nu )/B_j$, ($j=1,\ldots ,m$; $\nu =0, 1, 2, \ldots$), of the gamma functions $\Gamma (b_j+ B_j s)$ from the poles $(1-a_{\lambda } +k)/A_{\lambda }$, ($\lambda =1,\ldots ,n$; $k=0, 1, 2, \ldots$) of the gamma functions $\Gamma (1-a_{\lambda } - A_{\lambda } s)$, that is $A_{\lambda } (b_j+ \nu ) \ne B_j (a_{\lambda - k - 1})$. An empty product in 35, if it occurs, is taken to be one.

Detailed and comprehensive accounts of the matter are available in Mathai, Saxena, and Haubold^43^, Mathai and Saxena^44^, and Kilbas and Saigo^45^. Back to Eq. (33), we have:

36 $$\begin{aligned} E^{(r)}_{\alpha ,\beta }(-z) = H^{1,1}_{1,2}\left[ z \bigr | {\begin{matrix} (-r, 1) \\ (0,1), (-\alpha r, \alpha ) \end{matrix}} \right] , \end{aligned}$$

knowing that the generalized Mittag–Leffler function in terms of the Mellin–Barnes integral representation is given by^25^:

37 $$\begin{aligned} E_{\alpha ,\beta }^{\gamma }(z) = \frac{1}{\Gamma (\gamma )}\frac{1}{2\pi i} \int _{\Omega } \frac{\Gamma (-\xi ) \Gamma (\gamma + \xi ) (-z)^{\xi } d\xi }{\Gamma ( \alpha \xi + \beta )} \end{aligned}$$

and thus:

38 $$\begin{aligned} E_{\alpha ,\beta }^{\gamma }(z) = H^{1,1}_{1,2}\left[ z \bigr | {\begin{matrix} (1-\gamma , 1) \\ (0,1), (1-\beta , \alpha ) \end{matrix}} \right] . \end{aligned}$$

The two-parameter Mittag–Leffler (Eq. 32) is obtained by setting $\gamma =1$ in Eq. (38). With this one can rewrite Eq. (33) in the form

39 $$\begin{aligned} \hat{\rho }(k,t) = \sum _{r=0}^\infty \frac{(-1)^r t^{\alpha r} }{r!} H^{1,1}_{1,2}\left[ k^2 t^\alpha \bigr | {\begin{matrix} (-r, 1) \\ (0,1), (-\alpha r, \alpha ) \end{matrix}} \right] . \end{aligned}$$

#### Solution in the real-time space

Now we invert the Fourier transform in Eq. (39). To do this, we note that $\hat{\rho }(k,t)$ is an even function of *k*. For an even function ${\hat{f}}(k) = {\hat{f}}(-k)$, the Fourier transform reduces to the Fourier cosine transform,

40 $$\begin{aligned} f(x) = \frac{1}{2\pi } \int \limits _{-\infty }^{\infty } {\hat{f}}(k) e^{-ikx} \, dk = \frac{1}{\pi } \int \limits _{0}^{\infty } {\hat{f}}(k) \cos (kx) \, dk. \end{aligned}$$

The inverse Fourier cosine transform can be calculated using the following relation for the cosine transform of the *H*-function^46^

41 $$\begin{aligned} \int \limits _0^{\infty } k^{\rho -1} \cos (kx) H^{m,n}_{p,q} \left[ a k^\mu \bigr | {\begin{matrix} (a_p, A_p) \\ (b_q,B_q) \end{matrix}} \right] \, dk = \frac{\pi }{x^\rho } H^{n+1,m}_{q+1,p+2} \left[ \frac{x^\mu }{a} \bigr | {\begin{matrix} (1-b_q, B_q),(\frac{1}{2}+\frac{\rho }{2},\frac{\mu }{2}) \\ (\rho ,\mu ), (1-a_p,A_p),(\frac{1}{2}+\frac{\rho }{2},\frac{\mu }{2}) \end{matrix}} \right] . \end{aligned}$$

Using the latter with $\rho =1$, $a = t^\alpha$, $\mu =2$, and *m*, *n*, *p*, *q*, $(a_p,A_p)$ and $(b_q,B_q)$ coefficients defined in Eq. (39), one can invert the Fourier transform in Eq. (39) to obtain

42 $$\begin{aligned} \rho (x,t)= & {} \frac{1}{\pi } \sum _{r=0}^\infty \frac{(-1)^r t^{\alpha r} }{r!} \int \limits _0^{\infty } \cos (kx) H^{1,1}_{1,2}\left[ k^2 t^\alpha \bigr | {\begin{matrix} (-r, 1) \\ (0,1), (-\alpha r, \alpha ) \end{matrix}} \right] \, dk \nonumber \\= & {} \frac{1}{|x|} \sum _{r=0}^\infty \frac{(-1)^r t^{\alpha r} }{r!} H^{2,1}_{3,3} \left[ \frac{x^2}{t^\alpha } \bigr | {\begin{matrix} (1, 1), (1+\alpha r, \alpha ), (1,1) \\ (1,2), (1+r, 1), (1,1) \end{matrix}} \right] . \end{aligned}$$

Next, using the following reduction formula^42^

43 $$\begin{aligned} H^{m,n}_{p,q}\left[ z \bigr | {\begin{matrix} (a_1, A_1),\ldots ,(a_p,A_p) \\ (b_1,B_1),\ldots ,(b_{q-1},B_{q-1}), (a_1,A_1) \end{matrix}} \right] = H^{m,n-1}_{p-1,q-1}\left[ z \bigr | {\begin{matrix} (a_2, A_2),\ldots ,(a_p,A_p) \\ (b_1,B_1),\ldots ,(b_{q-1},B_{q-1}) \end{matrix}} \right] , \end{aligned}$$

we can simplify Eq. (42) to

44 $$\begin{aligned} \rho (x,t) = \frac{1}{|x|} \sum _{r=0}^\infty \frac{(-1)^r t^{\alpha r} }{r!} H^{2,0}_{2,2} \left[ \frac{x^2}{t^\alpha } \bigr | {\begin{matrix} (1+\alpha r, \alpha ), (1,1) \\ (1,2), (1+r, 1) \end{matrix}} \right] . \end{aligned}$$

Finally, using the property of the *H*-function^42^,

45 $$\begin{aligned} z^\sigma H^{m,n}_{p,q}\left[ z \bigr | {\begin{matrix} (a_p,A_p) \\ (b_q,B_q) \end{matrix}} \right] = H^{m,n}_{p,q}\left[ z \bigr | {\begin{matrix} (a_p+\sigma A_p,A_p) \\ (b_q+\sigma B_q,B_q) \end{matrix}} \right] , \sigma \in \mathbb {C}, \end{aligned}$$

with $\sigma = -1/2$, we come to

46 $$\begin{aligned} \rho (x,t) = \sum _{r=0}^\infty \frac{(-1)^r t^{\alpha (r-\frac{1}{2})} }{r!} H^{2,0}_{2,2} \left[ \frac{x^2}{t^\alpha } \bigr | {\begin{matrix} (1+\alpha [r-\frac{1}{2}], \alpha ), (\frac{1}{2},1) \\ (0,2), (r+\frac{1}{2}, 1) \end{matrix}} \right] . \end{aligned}$$

Together, Eqs. (15), (24), (39) and (46) provide the solution to the time fractional equation (12) in four different spaces with respect to the density arguments, namely $x \leftrightarrow k$, and $t \leftrightarrow s$.

### Case with $0<\alpha < 1$, $0<\beta < 2$

The solution to the bi-fractional Eq. (3) with $0<\alpha < 1$, $0<\beta < 2$, $\theta =0$ in real-time space can be obtained similarly to the time-fractional equation (12). The Laplace-Fourier transformations of Eq. (3) with the conditions given in (4) is:

47 $$\begin{aligned} \hat{\tilde{\rho }}(k,s) = \frac{s^{\alpha -1}}{s^\alpha + 1 + k^\beta }. \end{aligned}$$

The result for $\rho (x,t)$ is found to be:

48 $$\begin{aligned} \rho (x,t) = \frac{1}{|x|}\sum _{r=0}^\infty \frac{(-1)^r t^{\alpha r}}{r!} H^{2,1}_{3,3} \left[ \frac{|x|^\beta }{t^\alpha } \bigr | {\begin{matrix} (1,1), (1+\alpha r, \alpha ), (1,\frac{\beta }{2}) \\ (1,\beta ), (r+1, 1), (1,\frac{\beta }{2}) \end{matrix}} \right] . \end{aligned}$$

Using Eq. (45) with $\sigma = -1/\beta$, one can rewrite (48) as

49 $$\begin{aligned} \rho (x,t) = \sum _{r=0}^\infty \frac{(-1)^r t^{\alpha (r - \frac{1}{\beta })}}{r!} \times H^{2,1}_{3,3} \left[ \frac{|x|^\beta }{t^\alpha } \bigr | {\begin{matrix} (1-\frac{1}{\beta },1), (1+\alpha [r - \frac{1}{\beta }], \alpha ), (\frac{1}{2}, \frac{\beta }{2}) \\ (0,\beta ), (r+1-\frac{1}{\beta }, 1), (\frac{1}{2},\frac{\beta }{2}) \end{matrix}} \right] . \end{aligned}$$

## Numerical results

We calculate the obtained solutions for $\rho (x,t)$ governed by Eq. (3) with the boundary and initial conditions given by (4) for the four cases of (i) normal electrodiffusion ($\alpha =1$, $\beta =2$), (ii) time-fractional electrodiffusion ($0<\alpha <1$, $\beta =2$), (iii) space-fractional electrodiffusion ($\alpha =1$, $0<\beta <2$) and (iv) bi-fractional electrodiffusion ($0<\alpha <1$, $0<\beta <2$) as given by Eq. (49). We fixed the upper limit of the summation to five terms, which is deemed sufficient to represent well enough the overall behavior of the variable $\rho (x,t)$. The Fox *H*-function can be calculated numerically using a simple rectangular approximation of the integrals^47^. The function $\rho (x,t)$ is calculated for $x\in [-1,-\delta ) \cup (\delta ,1]$ and $t\in (\varepsilon ,0.25]$, where $\delta >0$ and $\varepsilon >0$ are utilized to cut small locality around $x=0$, $t=0$, where the Fox *H*-function and $\rho (x,t)$ are not defined. We remind again that $\rho (x,t)$ described by Eq. (3) is a generalization of the integer-order Debye–Falkenhagen approximation (Eq. (2)), whose validity is limited to the regime of small applied potentials.

First we consider the known integer-order case of $\alpha =1$, $\beta =2$ (i.e. Eq. (2)). It is clear that at the limit $\alpha \rightarrow 1$ we obtain from Eq. (46) the following expression for $\rho (x,t)$:

50 $$\begin{aligned} \rho (x,t) = \frac{e^{-\sqrt{\frac{x^2}{t}}}}{2 \sqrt{t}} + (-\sqrt{t} +\frac{t^{3/2}}{2} - \ldots ) H^{1,0}_{1,1} \left[ \frac{x^2}{t} \bigr | {\begin{matrix} (\frac{1}{2},1) \\ (0,2) \end{matrix}} \right] \end{aligned}$$

**Figure 2:**
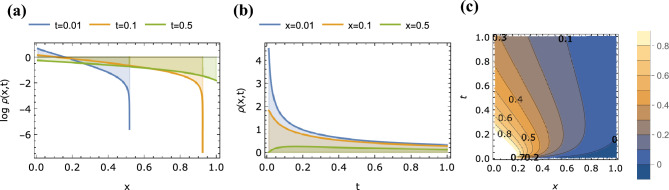
Plots of $\rho (x,t)$ given by Eq. (49) with $\alpha =1.0$, $\beta =2.0$ as a function of (**a**) *x* for $t=0.01$, 0.1, 0.5, (**b**) *t* for $x=0.01$, 0.1, 0.5 and (**c**) *x* and *t* (contour plot).

The same can be found from Eq. (49) for $\alpha \rightarrow 1$, $\beta \rightarrow 2$. We recognize that the first term in Eq. (50) corresponds to the fundamental solution of the standard Fick’s diffusion equation $\partial _t \rho = \partial _x^2 \rho$. Solutions to the integer-order case of Debye–Falkenhagen equation for different conditions has been previously provided mainly via numerical simulations and approximations (e.g. by using Padé approximation)^4,48,49^, but here by using tools from fractional calculus we give an analytical expression as an infinite series of the Fox *H*-function. Plots of $\rho (x,t)$ for this case as a function of *x* ($0.01<x<1$) for the different values of $t=0.01$, 0.1, 0.5 (in log-linear scale), and as a function of *t* ($0.01<t<1$) for the different values of $x=0.01$, 0.1, 0.5 (in linear-linear scale) are shows in Fig. 2a,b respectively. Figure 2c is the contour plot of $\rho (x,t)$ depicting its spatiotemporal dynamics. The solution depicting concentrations is always positive. It is an even function of *x* and decays to zero for large values of |*x*|. It also decays to zero for large values of *t*.

**Figure 3:**
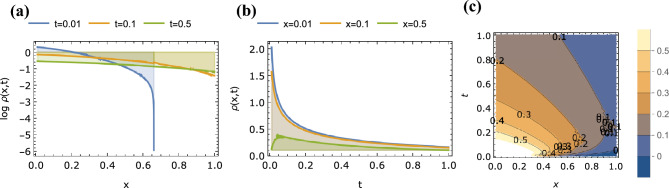
Plots of $\rho (x,t)$ given by Eq. (49) with for $\alpha =0.8$, $\beta =2.0$ as a function of (a) *x* for $t=0.01$, 0.1, 0.5, (b) *t* for $x=0.01$, 0.1, 0.5 and (c) *x* and *t* (contour plot).

**Figure 4:**
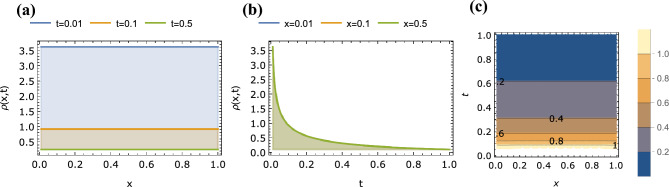
Plots of $\rho (x,t)$ given by Eq. (49) with for $\alpha =1.0$, $\beta =1.8$ as a function of (**a**) *x* for $t=0.01$, 0.1, 0.5, (**b**) *t* for $x=0.01$, 0.1, 0.5 and (**c**) *x* and *t* (contour plot).

**Figure 5:**
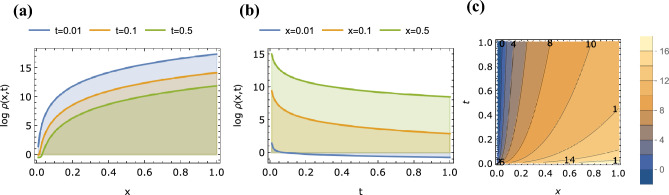
Plots of $\log (\rho (x,t))$ given by Eq. (49) with for $\alpha =0.8$, $\beta =1.8$ as a function of (**a**) *x* for $t=0.01$, 0.1, 0.5, (**b**) *t* for $x=0.01$, 0.1, 0.5 and (**c**) *x* and *t* (contour plot).

For the time-fractional anomalous case of $0<\alpha < 1$, $\beta =2$, we verify that Eq. (49) reduces to Eq. (46). Similar to the previous case, plots of $\rho (x,t)$ as a function of *x*, as a function of *t*, and as a function of both *x* and *t* for $\alpha =0.8$, $\beta =2$ are shown in Fig. 3.

For the space-fractional anomalous case of $\alpha = 1$, $0<\beta <2$, Eq. (49) simplifies to:

51 $$\begin{aligned} \rho (x,t) = (t^{-1/\beta } -t^{1-1/\beta } + \frac{1}{2} t^{2-1/\beta } -\ldots ) \times H_{2,2}^{1,1} \left[ \frac{x^{\beta }}{t} \bigr | {\begin{matrix} (1-\frac{1}{\beta },1), (\frac{1}{2}, \frac{\beta }{2}) \\ (0,\beta ), (\frac{1}{2}, \frac{\beta }{2}) \end{matrix}} \right] \end{aligned}$$

which is plotted in Fig. 4 for the case of $\alpha = 1$, $\beta =1.8$.

Finally, in Fig. 5 we show the variation of $\log \rho (x,t)$ vs. both variables *x* and vs. *t* for the general case of two fractional parameters, $\alpha = 0.8$ and $\beta =1.8$. The propagating quantity $\rho (x,t)$ tends to accelerate as *x* and *t* increase, and thus the representation in log scale.

## Conclusion

The traditional mathematical tools used for the modeling of transport in electrified porous structures in contact with an electrolyte are based on integer-order differential equations, which are more suited for homogeneous systems with planar geometries. When complex structures and coupled phenomena are involved, it is often required to further complement the existing models by additional approximations and assumptions which makes the problem even more difficult to solve. The theoretical and numerical results presented in this work show the possibilities that come with the use of both time and space bi-fractional-order derivatives for the case of the Debye–Falkenhagen equation, which is a simple and idealized model for electrodiffusion at low applied voltages. Eq. (49), with its extra two degrees of freedom $\alpha$ and $\beta$, compared to the integer-order model (Eq. (50)) is capable of deforming the spatiotemporal dynamics of the propagating quantity $\rho (x,t)$ in ways to account for subdiffusive and superdiffusive transports. While the physical interpretations of the fractional parameters remains unclear and need further studies, the mathematical solutions to this general problem can provide useful insights in anomalous transports in heterogeneous media such as membranes, protein channels and electrochemical devices.

## Data Availability

All data generated or analysed during this study are included in this published article
